# The Role of “Mixed” Orexigenic and Anorexigenic Signals and Autoantibodies Reacting with Appetite-Regulating Neuropeptides and Peptides of the Adipose Tissue-Gut-Brain Axis: Relevance to Food Intake and Nutritional Status in Patients with Anorexia Nervosa and Bulimia Nervosa

**DOI:** 10.1155/2013/483145

**Published:** 2013-09-09

**Authors:** Kvido Smitka, Hana Papezova, Karel Vondra, Martin Hill, Vojtech Hainer, Jara Nedvidkova

**Affiliations:** ^1^Institute of Endocrinology, Laboratory of Clinical and Experimental Neuroendocrinology, Narodni 8, 116 94 Prague 1, Czech Republic; ^2^Psychiatric Clinic, First Faculty of Medicine, Charles University, Ke Karlovu 11, 121 08 Prague 2, Czech Republic

## Abstract

Eating disorders such as anorexia (AN) and bulimia nervosa (BN) are characterized by abnormal eating behavior. The essential aspect of AN is that the individual refuses to maintain a minimal normal body weight. The main features of BN are binge eating and inappropriate compensatory methods to prevent weight gain. The gut-brain-adipose tissue (AT) peptides and neutralizing autoantibodies play an important role in the regulation of eating behavior and growth hormone release. The mechanisms for controlling food intake involve an interplay between gut, brain, and AT. Parasympathetic, sympathetic, and serotoninergic systems are required for communication between brain satiety centre, gut, and AT. These neuronal circuits include neuropeptides ghrelin, neuropeptide Y (NPY), peptide YY (PYY), cholecystokinin (CCK), leptin, putative anorexigen obestatin, monoamines dopamine, norepinephrine (NE), serotonin, and neutralizing autoantibodies. This extensive and detailed report reviews data that demonstrate that hunger-satiety signals play an important role in the pathogenesis of eating disorders. Neuroendocrine dysregulations of the AT-gut-brain axis peptides and neutralizing autoantibodies may result in AN and BN. The circulating autoantibodies can be purified and used as pharmacological tools in AN and BN. Further research is required to investigate the orexigenic/anorexigenic synthetic analogs and monoclonal antibodies for potential treatment of eating disorders in clinical practice.

## 1. Introduction

Anorexia nervosa (AN) and bulimia nervosa (BN) are eating disorders characterized by loss of self-control in eating behavior and disturbed emotions including high anxiety. These disorders affect 2-3% of young women [1]. AN is a serious eating disorder with the highest mortality rate among other psychiatric disorders [2, 3]. AN is characterized by chronic self-starvation, amenorrhea, and severe weight loss due to reduction of both fat mass and fat-free mass mainly at the expense of adipose tissue (AT). BN is an eating disorder in which the subject engages in recurrent binge eating. To compensate for the intake of the food and prevent weight gain, this is followed by induction of vomiting, use of laxatives, enemas, diuretics, excessive exercising, or fasting; this results in dysregulation of endogenous endocrine axes. In BN, the phenomenon of binge eating, that is, consumption of large amounts of food in a short time period, is accompanied by a sensation of losing control over eating [4]. 

**Figure 1:**
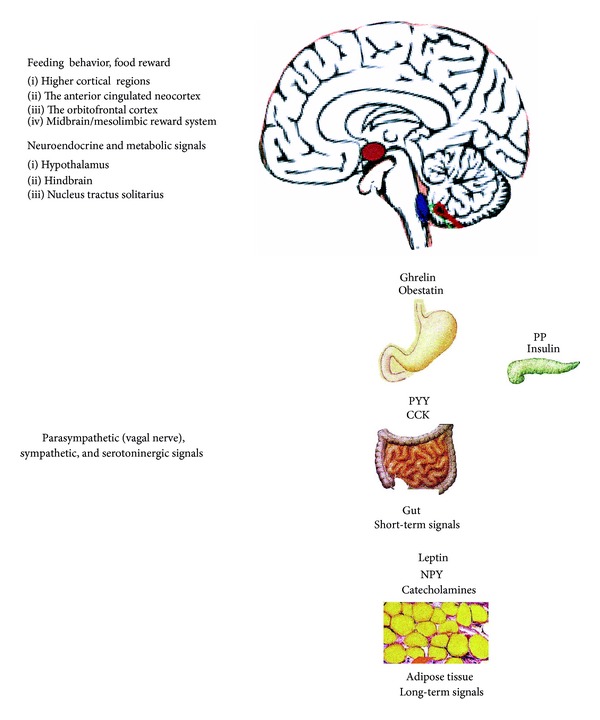
The role of adipose tissue- (AT-) gut-brain axis peptides in long-term and short-term regulation of food intake. Long-term regulators are adipose-derived food intake-inhibiting hormone leptin or food intake-stimulating hormone neuropeptide Y (NPY) mainly produced by the hypothalamus and also cosecreted with synthesized catecholamines in AT. Hormones produced in the gut are short-term food intake-stimulating hormone ghrelin, or food intake-inhibiting peptide YY (PYY), pancreatic polypeptide (PP), cholecystokinin (CCK), insulin, and putative anorexigen obestatin (the hypothalamus (violet), nucleus tractus solitarius (NTS, blue), sympathetic and serotoninergic areas (red), and vagal nerve parasympathetic area (green)).

The gastrointestinal tract, central nervous system, and AT referred to as the AT-gut-brain axis produce a series of hormones with orexigenic and anorexigenic effects [5–14] (Figure 1). On the one hand, ghrelin could represent a regulatory circuit controlling appetite and energy homeostasis by stimulating the release of other orexigenic peptides and neurotransmitters as well as neuropeptide Y (NPY) [15]. On the other hand, anorexigenic cholecystokinin (CCK), peptide YY (PYY), leptin, and putative anorexigenic hormone obestatin have an opposite effect at the hypothalamic level [16, 17]. The differential release of these hormones may act to initiate, maintain, or exacerbate cycles of food restriction or binge-purge behavior observed in AN and BN [18] (Tables 1 and 2). The former observations and recent reviews have suggested that AN and BN are linked to disturbed dopamine and serotonin systems [3, 19–23] which are related to anxiety, mood, and impulse control in patients with AN and BN. The inverse relationship between brain serotonin system and plasma ghrelin levels, hypothalamic NPY, and cocaine-amphetamine-regulated transcript (CART) expression in the regulation of feeding behavior was described in mice [24]. 

The abnormal eating behavior resulting in weight loss or weight gain contributes to an imbalance of energy metabolism and *in vivo* altered lipolysis and lipogenesis. Bradley et al. [25] hypothesized that orexigenic (appetite stimulating) neuropeptides promote positive energy balance and may potentially have antilipolytic properties, whereas anorexigenic (appetite-suppressing) neuropeptides promote weight loss and may stimulate lipolysis. The role of neuropeptides in mediating lipolysis and lipogenesis in humans is not well understood. Furthermore, sympathetic nervous system (SNS) and its neurotransmitter norepinephrine (NE) play a major role in regulation of AT lipolysis, appetite, energy expenditure, and the secretion of adipocytokines [26–30]. Very recently, it was revealed that the sympathetic innervation of AT is not only a source of catecholamines because adipocytes have the capacity to produce both NE and epinephrine [31] and that various stressors are able to stimulate production of catecholamines in adipocytes [32].

**Figure 2:**
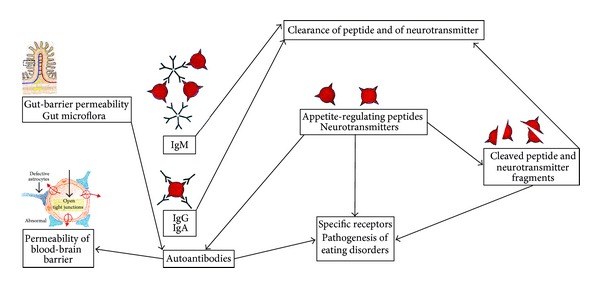
The role of up- or downregulated neutralizing autoantibodies (immunoglobulin (Ig) M, IgG, and IgA classes, and changes of their affinity) directed against appetite-regulating neuropeptides and peptides and neurotransmitters (dopamine, dopamine-beta-hydroxylase, and serotonin) in neuropeptidergic transmission and the pathogenesis of eating disorders. Producing excess of free fatty acids (FFA) and ketones to increase the permeability of the blood-brain barrier and to enter the cerebral matter in AN and BN [103]. Starvation, stress, catecholamines, microbial antigens, poststreptococcal autoimmune process (PANDAS), and proinflammatory cytokines decrease blood-brain barrier integrity in parallel with decreased levels of the tight junction protein, occludin [104]. Also autoantibodies against appetite-regulating peptides and neurotransmitters may disrupt the blood-brain barrier and the gut-barrier permeability in AN and BN [268]. Furthermore, gut-related antigens including gut microflora may influence production of specific autoantibodies (IgA class) against appetite-regulating hormones [47]. Indeed, starvation decreases the gut-barrier permeability in AN [102] and may decrease ghrelin autoantibodies (IgM, IgG, and IgA classes) production. However, realimentation-induced changes in the gut-barrier permeability and new antigenic stimulation during refeeding were accompanied by an increase of acylated ghrelin autoantibodies (IgM class) in AN [48].

In our previous studies, we observed *in vivo* increased SNS activity in subcutaneous abdominal AT in AN and BN patients [6, 33–36]. The cause and pathogenesis of AN and BN, however, remain unknown. The existence of a complex neurotransmitter-neuropeptide pathology in AN and BN could explain the pathogenesis of individuals with the eating disorder [21–23, 37–42]. From the point of view of etiopathogenesis of AN and BN, it would be of interest to study the autoantibodies that react with neuropeptides and neurotransmitters which play an important role in eating behavior, appetite control, and immunoregulatory system in AN and BN. Indeed, Corcos et al. [43] hypothesized that dopamine, dopamine-beta-hydroxylase, and serotonin acting with autoantibodies could be the antigenic cerebral targets reacting with “anti-brain” antibodies in BN. All autoantibodies against dopamine, dopamine-beta-hydroxylase (i.e., the enzyme that synthesizes NE from dopamine), and serotonin were lower in BN than in the controls (Table 1). Moreover, the autoantibodies directed against feeding-stimulatory and feeding-inhibitory neuropeptides have been reported in patients with AN and BN. These autoantibodies correlated with psychopathological traits in individuals with eating disorders [44–46] and that neutralizing autoantibodies directed against appetite-regulating peptides were classified as important attributors to mechanisms controlling motivation in AN and BN (Figure 2). Fetissov et al. [47] studied healthy women for the presence of autoantibodies directed against 14 key appetite-regulating neuropeptides or peptide hormones including NPY, ghrelin, leptin, or PYY (Tables 1 and 2). Thus, these results confirmed that autoantibodies against hormones represent a general physiological phenomenon, suggesting its implication in physiological peptidergic transmission. The detection of the immunoglobulin (Ig) A class of such autoantibodies supports the antigenic stimulation by gut microflora in healthy subjects [47] (Figure 2). In fact, patients with AN display increased plasma levels of alpha-melanocyte-stimulating hormone (*α*-MSH) autoantibodies [45] contrasting with lower levels in acylated ghrelin autoantibodies [48] (Table 1). The presence of immune complexes sequesters autoantibodies against nonacylated ghrelin resulting in higher levels of free acylated ghrelin in AN patients, and eventually resulting in ghrelin resistance in AN [48]. Intriguingly, this is a potentially analogous situation in which autoantibodies against insulin may play a role in the shifts of bioavailable levels of insulin with possible effects on hypoglycemia. Involvement of insulin autoantibodies in insulin resistance has been extensively studied as a mechanism underlying insulin resistance after insulin administration [49] and autoantibodies against insulin have been studied as a marker of type 1 diabetes [50]. Using homeostasis model assessment of insulin resistance (HOMA-IR), we found significantly lower values of HOMA-IR in malnourished and underweight AN patients when compared with the controls [28, 29]. However, refeeding is associated with the onset of insulin resistance in AN patients [51].

The aim of this review was to describe the key role of orexigenic and anorexigenic hormones originating from the gut, central nervous system, and AT and to discuss how an impairment of energy balance and interaction between these factors and up- or downregulated neutralizing autoantibodies are involved in the pathogenesis, autoimmunity, regulation of food intake, energy expenditure, and growth hormone (GH) release in eating disorders. Understanding of the pathogenic mechanisms may contribute to more specific and effective therapy in AN and BN. 

## 2. An Overview of Hunger-Satiety Signals and Autoantibodies against Appetite-Regulating Neuropeptides and Peptides of the AT-Gut-Brain Axis 

### 2.1. Ghrelin

Ghrelin is a 28-amino acid peptide which increases food intake and acts as an endogenous stimulator of GH [52]. The ghrelin gene encodes a polypeptide preproghrelin containing 117 residues which undergoes stepwise processing to form ghrelin [53]. Ghrelin is predominantly produced by the stomach but is also expressed in many other tissues and is the first identified hormone containing acylated n-octanoic acid in its residues by ghrelin-O-acyl-transferase (GOAT) [54, 55]. This acylation is essential for binding to the growth hormone secretagogue (GHS) receptor type 1a (GHS-R 1a) and for the GH-releasing and appetite-stimulating activities. Genetic variation of GOAT is implicated in the etiology of AN [56]. Unexpectedly to the ghrelin secretion pattern, it was shown that GOAT mRNA levels decrease upon prolonged food deprivation and increase postprandially. Therefore, GOAT-ghrelin system may also act as a nutrient sensor by using readily absorbable medium-chain fatty acids to signal to the brain that high caloric food is available [57] and that dietary lipids can directly influence ghrelin acylation. 

The effect of ghrelin on GH release is two to three times greater than that of GH-releasing hormone (GHRH) in humans [58]. Moreover, peripherally administered ghrelin signals *via* the vagus nerve to the brain where it triggers the release of GHRH and contributes to the activation of the food intake signalling cascade by NPY neurons in the arcuate nucleus of the hypothalamus [59]; when the vagus nerve is cut, the induction of GH release after ghrelin injection is dramatically decreased. It was documented that GH inhibits stomach ghrelin secretion. These findings indicate that the vagal circuit between the brain and the stomach has a crucial role in regulating plasma ghrelin levels [60]. Asakawa et al. [61] indicated that in contrast to acylated ghrelin, nonacylated ghrelin induces a negative energy balance by decreasing food intake and delaying gastric emptying. Nonacylated ghrelin present in plasma in far greater quantities than acylated ghrelin seems to be devoid of any endocrine action. However, it is able to exert some nonendocrine actions including cardiovascular and antiproliferative effects by binding different GHS-R subtypes [62]. It was described that acylated ghrelin [63] and nonacylated ghrelin pass the blood-brain barrier by means of transmembrane diffusion [64]. Nonacylated ghrelin induces an increase in neuronal activity in the arcuate nucleus, is involved in the regulation of the synthesis of anorexigenic mediators like urocortin and CART in the hypothalamus, and interacts with the corticotropin releasing factor receptor subtype 2 (CRF_2_-R). Therefore, the neuromodulatory peptides CART and urocortin might thus play a key role in the anorexigenic effect of nonacylated ghrelin *via* CRF_2_-R-dependent signalling [65].

The role of acylated ghrelin, nonacylated ghrelin, and other ghrelin gene-derived peptides in the postprandial regulation of satiety was not established. Recently, we found decreased levels of plasma total, acylated, and nonacylated ghrelin and obestatin after a high-carbohydrate breakfast in healthy women [66]. It is possible that obestatin may postprandially blunt the effect of ghrelin in healthy normal weight women. 

Ghrelin increases food intake through effects on NPY [67]. Recently, studies in rodents suggested a possible mediation of ghrelin action on GH by NPY and that GH may be involved in maintaining feeding [68]. Plasma ghrelin levels are elevated during fasting and suppressed after meal. It was shown that the efferent vagus nerve contributes to the fasting-induced increase in ghrelin secretion and that higher ghrelin stimulates the afferent vagus nerve, promotes food intake, and contributes to ghrelin-induced GH secretion. These findings demonstrate that the vagal circuit between the brain and stomach has an important role in regulating plasma ghrelin levels [60]. Peripheral ghrelin signalling, which travels to the nucleus tractus solitarius (NTS) in part *via* the vagus nerve, increases NE in the arcuate nucleus of the hypothalamus, thereby stimulating feeding, at least partially through alpha_1_- and beta_2_-noradrenergic receptors [69]. Furthermore, ghrelin may have an integrative role in the behavioral and an adaptive response to starvation by increasing anxiety and alertness in animals and humans [70]. Ghrelin is also a potent secretagogue for GH and an intravenous (i.v.) ghrelin administration stimulates GH release in a dose-dependent fashion in humans [71], and there may be a positive association mediated by ghrelin, alternatively, a negative feedback action such that inhibition of plasma ghrelin levels occurs when plasma GH levels are high [72]. 

Fasting plasma ghrelin concentrations in humans are negatively correlated with body mass index (BMI) [73]. In obese individuals, dieting is associated with an increase in plasma ghrelin levels [74]. Both peripherally and centrally administered ghrelins produce a positive energy balance and lead to body weight gain [75]. In humans, acylated ghrelin induces a rapid rise in blood glucose and plasma insulin levels. However, coadministration of nonacylated ghrelin counteracts this effect. A separate i.v. administration of nonacylated ghrelin improves glucose metabolism and insulin sensitivity and inhibits lipolysis in humans [76]. Based on these data, Van Der Lely [76] suggests the existence of a specific receptor for nonacylated ghrelin other than CRF_2_-R and GHS-R 1a. 

Current analysis has the expression of ghrelin in a number of endocrine tissues such as AT [77]. Recently, Liu et al. [78] have explored the effects of ghrelin on the proliferation and differentiation of preadipocytes *in vitro* and confirm that ghrelin induces the differentiation of 3T3-L1 preadipocytes into mature adipocytes. Rodent and human studies indicate that ghrelin elicits an antilipolytic effect mediated by both acylated and nonacylated ghrelin and promotes adipogenesis [79]. However, predominant nonacylated ghrelin does not appear to activate GHS-R1a, and it remains unclear through which receptor nonacylated ghrelin mediates its action in AT, although it has been suggested that the antilipolytic effect of ghrelin could be mediated by an unidentified non-GHS-R 1a receptor [80]. Interestingly, the ratio of acylated and nonacylated ghrelin production might help to regulate the balance between adipogenesis and lipolysis in response to nutritional status [81]. Recently, Tebbe et al. [82] have shown that ghrelin effects in the rat central nervous system appear to be mediated through receptor Y1, which also mediates the antilipolytic action of NPY_1–36_ and PYY_1–36_. Thus, ghrelin may mediate its peripheral action in AT through Y1 receptor. Kos et al. [83] have demonstrated antilipolytic action of ghrelin in human AT and showed that acylated and nonacylated ghrelin may be ligands for Y1 mediating lipogenic effect in humans.

#### 2.1.1. Ghrelin Levels and Autoantibodies against Ghrelin before and after Realimentation in AN and BN

Fasting plasma ghrelin levels have been reported to be increased in underweight patients with AN, especially in patients with binge-purge subtype of AN as compared to patients with restrictive type of AN, suggesting that binge-purging behavior has some influence on plasma ghrelin [84, 85] (Table 1). These findings were not confirmed by Otto et al. [86], who did not find difference in plasma ghrelin between restrictive and binge-purge subtypes of AN, and by Troisi et al. [87], who detected opposite results with higher plasma ghrelin levels in restrictive type of AN as compared to patients with binge-purge subtype of AN and BN individuals. Ghrelin is increased in the case of AN, and this increase in plasma ghrelin levels may occur either as an adaptive response to correct the abnormal energy status or as a result of relative resistance to ghrelin [88].

A greater fall of plasma ghrelin levels was seen in AN than normal controls following an euglycemic hyperinsulinemic clamp [89]. In women with AN, Karczewska-Kupczewska et al. [89] reported significant positive correlation between fasting ghrelin and insulin sensitivity and that the progressive decline in circulating insulin would favor ghrelin production in AN [84]. The enhanced plasma ghrelin levels of underweight AN patients tend to normalize after refeeding [90]. Furthermore, patients with AN do not show a decrease in plasma ghrelin following a standardized meal that is observed in healthy women [91], and anorectic patients would be refractory to the orexigenic action of ghrelin to regain a normal weight and replenish energy stores (Table 1). 

Furthermore, our group assumes that the preproghrelin is cleaved differently in eating disorders such as AN than under physiological conditions. Thus, intact acylated ghrelin 1–28 is rapidly degraded to nonacylated forms or smaller fragments in AN patients. Alternatively, a loss of function mutations in GOAT might disturb the ratio of acylated ghrelin to nonacylated ghrelin [56]. These hypotheses are in keeping with the finding of Hotta et al. [92] who reported decreased levels of plasma acylated ghrelin in AN patients. However, Germain et al. [93] documented that the acylated ghrelin/total ghrelin ratio has been found to be increased in the restrictive type of AN, whereas it decreased in the binge-purge type of AN and BN.

GH levels are higher in patients with AN than in controls, and these higher levels are consequent to higher levels of ghrelin, a GH secretagogue. Thus, the hypersecretion of ghrelin might contribute to the hypersomatotropism of AN [94]. Indeed, a dysfunction of the ghrelin feedback systems might lead to the pathophysiology of AN and BN [60]. Furthermore, Støving et al. [95] suggest that GH hypersecretion in AN is due to decreased hypothalamic somatostatinergic tone restored by weight gain in these patients. 

The physiological inhibitory role of free fatty acids (FFA) on GH secretion seems to be preserved in patients with AN. In fact, the infusion of FFA inhibited the elevated basal GH levels and abolished the exaggerated GH response to the GHRH, whereas the administration of antilipolytic drug Acipimox (Aci) led to the decrease in plasma FFA and markedly enhanced the GHRH-induced GH rise in patients with AN but not in healthy women [96]. Although patients with AN showed a hyperresponsiveness to GHRH administration [95], their GH response to ghrelin administration is surprisingly blunted [97]. This finding is consistent with desensitization of the GHS receptor induced by the chronic elevation of ghrelin levels in AN or impaired metabolic status in AN because ghrelin administration was not followed by increase in blood glucose levels in these patients [97]. Indeed, AN is associated with a nutritionally acquired resistance to GH with elevated GH levels, and low levels of the GH-binding protein indicate decreased expression of the GH receptor, which accounts for the state of GH resistance in the starved state [98]. This is consistent with results reported by Fazeli et al. [99] that administration of supraphysiological recombinant human GH in patients with AN does not overcome the state of GH resistance. Therefore, the administration of recombinant human GH was not associated with a significant change in plasma levels of blood glucose, insulin, or FFA in AN. Importantly, these findings suggest that patients with AN would have a relatively high resistance to the effects of GH [99]. In AN, a greater mobilization of FFA leads to an increase in the peroxisome proliferator-activated receptor-alpha (PPAR-*α*) which increases levels of fibroblast growth factor 21 (FGF 21) [100]. In fact, FGF 21 is a novel adipocytokine which may mediate GH resistance and reduces insulin growth factor 1 (IGF-1) levels in AN [101]. Patients with AN display low levels of autoantibodies against acylated ghrelin and higher levels of autoantibodies against nonacylated ghrelin present as immune complexes. Interestingly, the negative correlations were found between plasma autoantibodies and ghrelin peptides, and the decrease of bioavailable ghrelin autoantibodies may underlie an increase of plasma ghrelin levels and the resulting phenomenon of ghrelin resistance in patients with AN [48]. Indeed, starvation-induced changes decreased gut-barrier permeability [102] and may decrease ghrelin autoantibodies (IgM, IgG, and IgA classes) in AN. However, Terashi et al. [48] found that refeeding in AN patients was accompanied by an increase of acylated ghrelin autoantibodies (IgM class), which may indicate new antigenic stimulation leading to realimentation-induced changes in the gut-barrier permeability in AN patients (Table 1, Figure 2). In contrast to the decreased gut-barrier permeability during starvation in AN, increased levels of plasma FFA and the more ketone bodies produced increase the permeability of the blood-brain barrier during starvation and weight loss in patients with AN [103]. Moreover, starvation, stress, catecholamines, microbial antigens, poststreptococcal autoimmune process (PANDAS), and proinflammatory cytokines decrease blood-brain barrier integrity in parallel with decreased levels of the tight junction protein, occludin [104] (Figure 2). Hence, it is possible that access of high affinity autoantibodies against appetite-regulating neuropeptides and peptides in the brain centers normally protected by the blood-brain barrier may trigger the development of AN and BN (Figure 2). 

 It is accepted that plasma levels of active acylated ghrelin represent less than 10% of circulating total ghrelin levels, which include acylated and inactive nonacylated ghrelin. We found increased plasma nonacylated ghrelin but not acylated ghrelin levels in AN patients (unpublished data). While high plasma total ghrelin in AN has been consistently observed [91, 105], elevated acylated ghrelin was found in few studies [106, 107]. 

It was reported that fasting plasma ghrelin was higher in the purging type of BN in comparison to the nonpurging type and in comparison to controls [108, 109]; this supports the idea that binge-purge cycles have an influence on fasting plasma ghrelin. However, subsequent studies did not detect any significant difference in plasma ghrelin levels between binge-purge BN patients and controls [12, 13, 87, 110] though Kojima et al. [111] found that BN patients exhibited elevated ghrelin levels despite higher BMI. In our recent studies, we reported increased response of GH and ghrelin to short-term exercise and antilipolytic drug Aci in BN patients and confirmed that GH exerted an inhibitory feedback effect on plasma ghrelin during exercise only in BN patients but in both BN patients and healthy women during exercise with Aci administration [12, 13]. Therefore, these data established ghrelin as a potential discriminator between women with eating disorders and healthy women [87]. Furthermore, the ghrelin responses to a standardized meal have been reported to be blunted in symptomatic binge-purge BN patients as compared to healthy controls [110–112]. However, in our recent study, we documented decreased ghrelin levels in BN patients after a high-carbohydrate breakfast [11] (Table 1). 

In contrast with anorectic patients, the normal GH response to ghrelin administration was observed in BN patients, and ghrelin administration was followed by increase in blood glucose in BN [113]. These authors hypothesize that ghrelin hypersecretion may have a role in eating behavior but normal GH and blood glucose response to ghrelin administration may reflect less impaired nutritional status in BN patients [113]. 

### 2.2. Obestatin

Recently, it has been demonstrated that preproghrelin undergoes additional proteolytic cleavage, generating a 23-amino acid peptide, which has been named obestatin, and amidation of obestatin is likely essential for its biological activity as well as acylation of ghrelin. Responses to obestatin_1–23_ were greater than those to obestatin_1–10_ and obestatin_11–23_ [114]. Unlike ghrelin, treatment with obestatin did not increase GH secretion [17]. Interestingly, obestatin antagonized GH secretion and food intake induced by ghrelin only when ghrelin and obestatin were coadministered [115]. Fasting obestatin levels were significantly lower in obese patients than in normal weight and anorectic women [116], and significant increase of both plasma obestatin and ghrelin levels was demonstrated with weight loss in obese patients [117]. In contrast to ghrelin, obestatin has anorexigenic effects, reduces gastric emptying, inhibits jejunal contractions, and suppresses body weight gain [17]. However, several recent studies performed in rats and mice under various experimental conditions did not reproduce these results [118, 119] and did not support the concept that obestatin is an opponent or counterpart of ghrelin. Indeed, Gourcerol et al. [118] proposed to rename obestatin to ghrelin-associated peptide. It was revealed that the action of obestatin on the secretion of insulin, glucagon, and somatostatin is the same as the action of acylated ghrelin [120]. 

Further studies showed that obestatin was involved in inhibiting thirst and vasopressin secretion [121], affecting cell proliferation [122], increasing the secretion of pancreatic juice enzymes [123], and inhibiting glucose-induced insulin secretion [124]. Although Zhang et al. [17] implied that amidation of obestatin is essential for obestatin activity, Van Dijck et al. [119] did not demonstrate any suppressive effects on eating and drinking after central administration in mice despite using amidated obestatin. Interestingly, Pan et al. [125] reported that obestatin is unable to cross the blood-brain barrier and is rapidly degraded in the circulation; this was confirmed by Vergote et al. [126]. Therefore, an alternative hypothesis is that obestatin exerts its effects on eating and drinking through direct interactions with the gastrointestinal system. Indeed, Zhang et al. [17] observed decreased contractile activity of jejunum muscle strips *in vitro* and suppression of gastric emptying *in vivo* after obestatin treatment. Thus, the inhibition of jejunal contraction could generate an afferent vagal signal to induce satiety in the brain. Obestatin is an interesting peptide but controversial gut hormone. It was concluded that obestatin exerts a dual effect on glucose-induced insulin secretion. At a low glucose concentration, obestatin potentiated the insulin response to glucose. At a high glucose concentration, obestatin inhibited the insulin release [127]. 

In our previous study, Sedláčková et al. [66] demonstrated that plasma obestatin levels decrease similarly to ghrelin after a high-carbohydrate breakfast in healthy women. A possible explanation of the simultaneous postprandial decrease of obestatin with ghrelin is that the function of obestatin may be to antagonize orexigenic ghrelin action after food intake. Interestingly, obestatin was positively correlated to total ghrelin, nonacylated ghrelin, and NPY. The positive relationship of obestatin with total ghrelin in the postprandial period indicates that these two cleavage products of one gene could act in a similar fashion to increase food intake. This idea is confirmed by the positive correlation between obestatin and orexigen NPY. However, this positive relationship of obestatin with the nonacylated ghrelin may correspond with the idea of a dual effect of obestatin. 

Zhang et al. [17] reported that obestatin was the cognate ligand for the orphan G-protein-coupled receptor 39 (GPR39) based on the claim of its binding to human GPR39 with high affinity and specificity. However, recent reports indicate that obestatin is unlikely to be the endogenous ligand for GPR39 due to the lack of specific binding on GPR39 receptor-expressing cells and the absence of signal transduction pathway activation [128]. The native receptor for obestatin remains to be identified.

Recently, Zhang et al. [129] suggested that obestatin was a hormone capable of binding to GPR39 to regulate functions of gut and AT. Another observation revealed that the obestatin receptor GPR39 was upregulated in AT during fasting, whereas GPR39 levels were decreased in cultured mouse embryonic fibroblast cell lines (related to 3T3-L1) during adipogenesis [130]. A decreased obestatin receptor GPR39 expression in human AT was found in patients with obesity [131]. These findings suggest a possible role of the obestatin receptor GPR39 in adipogenesis. It was speculated that the obestatin receptor GPR39 could possibly play a similar role in the liver, adipose, endocrine pancreas, and gastrointestinal tract tissue regeneration and differentiation [132]. Furthermore, Granata et al. [133] documented that obestatin promotes beta cells and human islets survival by binding to glucagon-like peptide-1 (GLP-1), that is, the receptor via which incretins act. Very recently, Fujimiya et al. [134] supposed that obestatin may act on the obestatin receptor on vagal afferent nerve terminals, and CRF-R and urocortin-2 neurons in the hypothalamus may mediate the action of obestatin to inhibit the gastroduodenal motility via CRF_1_-R and CRF_2_-R in the brain.

#### 2.2.1. Obestatin Levels in AN and BN

Recently, Sedlackova et al. [11] documented that fasting plasma obestatin levels were increased in both AN and BN patients compared to controls (Table 2). Monteleone et al. [135] found that underweight AN patients displayed increased plasma obestatin and ghrelin levels and an increased ghrelin/obestatin ratio compared with healthy women, which may suggest that the hunger signal of ghrelin is stronger than the satiety signal of obestatin. A limitation of this study is represented by the low number of AN-restrictive patients with respect to AN binge-purge individuals because in AN binge-purge subjects ghrelin levels have been reported to be higher than in AN-restrictive ones [109]. In addition, Zamrazilová et al. [116] failed to reveal any significant differences in plasma obestatin levels between restrictive type of AN and normal weight women, but the higher ghrelin to obestatin ratio in AN might reflect a long-term reduction in energy intake which could contribute to susceptibility of AN women to bulimic episodes. No significant changes in these parameters were detected in BN patients [135]. Moreover, Harada et al. [107] and Nakahara et al. [136] showed increased plasma obestatin and ghrelin levels in small groups of AN restrictive patients compared with age-matched healthy women. None of these studies, however, calculated the ghrelin/obestatin ratio. 

Recently, Germain et al. [105] have reported increased plasma obestatin and ghrelin levels and decreased ghrelin/obestatin ratio in restrictive AN. The decreased ghrelin/obestatin ratio could facilitate the food intake restriction in these patients if obestatin inhibitory effects on food regulation were more validated.

However, Sedlackova et al. [11] reported that the administration of a high-carbohydrate breakfast induced a similar relative decrease in plasma ghrelin and obestatin in AN, BN patients and the controls suggesting a role of obestatin with rather orexigenic properties (Table 2). Moreover, we found that the ghrelin/obestatin ratio was lower in AN compared to BN and controls. We suggested that different plasma obestatin levels in AN and BN may have demonstrated their diverse function in eating behavior. Germain et al. [93] revealed that total and acylated ghrelin and obestatin circadian levels are increased in patients with AN restrictive type compared with the controls but decreased in patients with AN binge eating/purging type and those with BN. Recently, Uehara et al. [137] reported that an increase in energy intake leads to a decrease in plasma obestatin levels in patients with AN restrictive type (Table 2). 

### 2.3. NPY

NPY is a 36-amino acid peptide that has potent orexigenic properties [138]. Experimental evidence indicates that NPY is the strongest orexigenic factor in the hypothalamic control of feeding behavior [139]. NPY's activity in cellular metabolism is mediated through binding to G-protein-coupled receptors, of which at least four subtypes exist in humans (Y1, 2, 4, and 5) and which are present in most peripheral tissues. The hypothalamic Y1, Y2, Y4, and Y5 receptors have all been hypothesized to mediate the orexigenic effects of NPY [140]. 

NPY coexists with catecholamines in the central and SNS and in the adrenal medulla [141]. In coculture with adipocytes, sympathetic neurons secreted NPY, suggesting cross-talk between the neural cells and adipocytes [142]. Furthermore, NPY-containing nerves are present in the gut of many species. Orexigenic peptide NPY participates in ghrelin and GH regulation pathways [143]. Coiro et al. [144] revealed that a somatostatinergic pathway is involved in the mechanism connecting physical exercise to NPY secretion in humans. Even though plasma NPY levels do not reflect NPY secretion in the central nervous system, there is no clear evidence that plasma NPY levels originate from peripheral sympathetic nerve secretion or the adrenal gland and/or AT during exercise in humans [145]. 

The i.v. administration of NPY has no effect on GH secretion in healthy humans [146]. However, stimulatory effects of NPY on GH secretion have been reported in prolactinoma and acromegalic patients [147, 148]. In addition, some of these authors also described inhibition of GH secretion by NPY [148].

The role of NPY can be considered as helping to coordinate protective antistarvation activity and preventing further depletion of existing energy stores. Antilipolytic effect of NPY may also regulate plasma FFA. As FFA regulate insulin sensitivity, an impairment in NPY's antilipolytic action could lead to changes in insulin resistance [149]. 

Intracerebroventricular injection of NPY appears to mediate upregulation of the key enzyme of lipogenesis: lipoprotein lipase expression and activity in AT [150]. It was recently found that NPY is synthesized in human AT and stimulates the proliferation and differentiation of new adipocytes [151, 152]. Thus, AT-derived NPY could cause a significant rise of plasma NPY levels and may mediate reduction of leptin secretion [151]. Although to date the role of most of these receptors in human AT is poorly understood, binding studies [25] have suggested that Y1 receptor may mediate the antilipolytic effect of NPY in AT. NPY_1–36_ is cleaved by dipeptidyl peptidase IV (DPP-IV) to generate the truncated NPY_3–36_, with which DPP-IV diverts affinity of NPY from Y1 to other receptors such as receptor Y5 whose function remains elusive [153]. DPP-IV inhibitors are therefore likely to enhance the antilipolytic action of NPY_1–36_ as well as PYY_1–36_ [153]. Furthermore, in order to better understand the interactions between sympathetic neurotransmitters and glucocorticoids in AT, Kuo et al. [154] treated sympathetic neural cells with dexamethasone upon which the expression of NPY and its Y2 receptor was more than doubled. Therefore, cortisol and the adrenergic activity seem to converge on the NPY-Y2 adipogenic system. Thus, adipose-derived NPY may have implications for central feedback of adiposity signals.

#### 2.3.1. NPY Levels in AN and BN

NPY is one of the primary systems regulating the stress response, emotionality, and hormones relevant to AN and BN. It has been reported that NPY can attenuate specific behavior when the organism is stressed, and antistress effects of NPY are relevant to psychiatric conditions such as AN and BN [6, 155, 156] and that NPY has an anxiolytic and antidepressive behavior profile [157]. Interestingly, it is possible that NPY contributes to both binge eating and subsequent purging in BN because NPY itself has been demonstrated to induce an emetic response [158].

In reports with AN, basal plasma NPY levels in AN patients did not differ from the levels in the controls [159, 160]. Discordant data have been published concerning NPY levels in AN patients. Plasma levels of NPY were significantly lower in anorectic women than in the control group [161], and plasma NPY was decreased during treatment of anorectic girls. These changes do not correspond with increasing body weight suggesting dysregulation of appetite and body weight control mechanisms in AN [162]. The study by Sedlackova et al. [14] published during the preparation of this paper assumes that increased fasting NPY levels unchanged after a high-carbohydrate and high-protein breakfast indicate that NPY may be an important biomarker for disturbed eating behavior in AN and BN patients (Table 1). 

Recent studies have suggested that NPY is not merely an “orexigen” but acts to stimulate behavior which precedes the food intake and actually inhibits intake per se [163, 164]. It was found that the treatment with NPY increased physical activity, decreased food intake and caused a loss of body weight in rats [165]. From this point of view, it is possible that AN patients are physically hyperactive and eat only a little food in spite of having depleted body fat and pathologically upregulated hypothalamic orexigenic peptides [165].

Plasma levels of NPY during symptomatic and remission phases of BN are unchanged compared with age- and weight-matched controls [38]. However, plasma concentrations of NPY in patients with BN were significantly elevated in comparison to controls [6, 11, 13, 14, 161] (Table 1). In our recent study, we revealed that antilipolytic drug Aci during short-term exercise further increases plasma NPY levels in patients with BN [6]. 

### 2.4. PYY

PYY is a 36-amino acid gut peptide belonging to the same family as NPY, and PYY has recently been discovered in the hypothalamus of the human brain. PYY is released from the endocrine L cells of the distal ileum and colon in response to feeding [166]. PYY in the circulation exists in two major forms: PYY_1–36_ and PYY_3–36_. PYY_3–36_ binds with the greatest affinity at the presynaptic inhibitory Y_2_ receptor and is a peripherally active anorectic signal. PYY_3–36_ is the product of cleavage of the amino terminus residues by DPP-IV from PYY_1–36_. PYY is able to cross the blood-brain barrier by transmembrane diffusion from the circulation [167]. Evidence suggests that the anorectic effect of peripheral PYY_3–36_ may be mediated via the presynaptic inhibitory Y_2_ receptor present on arcuate NPY neurons [168]. It was also shown that PYY_3–36_ inhibited dopamine and NE release through the NPY Y2 receptors in the hypothalamus supporting a central anorectic effect of PYY_3–36_ [169]. In contrast to peripheral PYY_3–36_, centrally administered PYY_1–36_ and PYY_3–36_ increase food intake. PYY injection into the third, lateral, or fourth cerebral ventricles potently stimulates food intake in rodents [170]. Therefore, while circulating, PYY_3–36_ may access the higher affinity arcuate nucleus Y_2_ receptors, and the central feeding effects of PYY_1–36_ and PYY_3–36_ may be mediated by lower affinity Y_1_ and Y_5_ receptors [16]. Circulating PYY levels are low in the fasting state and rapidly increase postprandially when PYY is released into the circulation [171]. Either central or peripheral administration of PYY reduces food intake and body weight gain in humans [168]. The role of PYY in the regulation of energy balance in humans remains to be clarified; however, reduced caloric intake was demonstrated following infusion of PYY [168]. Moreover, PYY is known to cause nausea and emesis in some individuals [172, 173] which can contribute to subsequent self-induced vomiting in BN. A single infusion of PYY_3–36_ is capable of reducing food intake in lean and obese humans and decreasing circulating ghrelin levels [174]. Thus, it appears that peripheral PYY_3–36_ acts as a satiety signal regulating the termination of meal, partially by decreasing the production of the hunger-stimulating plasma ghrelin. In addition, in healthy subjects, there is a negative correlation between plasma levels of ghrelin, PYY, and BMI, respectively [111]. Also a negative association between PYY and leptin levels was described. It was suggested that PYY levels increase with weight loss and when plasma leptin is low [175]. 

It was shown that exercise can function as a physiological regulator of hormone release in appetite control and that levels of PYY_3–36_ are positively correlated with exercise intensity [176, 177].

Furthermore, the antilipolytic effect of PYY and NPY has been shown in human adipocytes. Labelle et al. [178] have noted that the receptor Y1 mediates the antilipolytic effect of NPY and PYY in rat adipocytes. Current evidence supports that Y1 does not only bind to NPY_1–36_ but can bind other ligands of the pancreatic polypeptide family with potentially higher binding affinities for PYY_1–36_ [83]. 

#### 2.4.1. PYY Levels in AN and BN

Studies of PYY_3–36_ secretion in AN and BN are still scanty [179]. In patients with AN, basal plasma PYY_3–36_ levels have been reported to be normal [180], increased [181–183] or reduced [184]. Moreover, in anorectic patients, plasma PYY_3–36_ response to food intake has been detected to be time delayed [180] or increased [182] (Table 2). After a partial body weight regain, the PYY_3–36_ response to a test meal was not completely restored in AN patients [182]. Recently, Sedlackova et al. [14] documented that basal plasma PYY levels were similar in AN and BN groups and reached significantly higher values after high-protein breakfast compared with high-carbohydrate breakfast suggesting an important role of ingested macronutrient in plasma levels of PYY (Table 2). 

In BN, basal plasma PYY levels increase to markedly high values during the phases of abstinence from binge eating and vomiting to return to control levels after recovery [185, 186]. However, we and the others found that fasting plasma PYY levels during symptomatic phase of BN were unchanged and comparable with age- and weight-matched healthy women [14, 187]. Recently, a blunted PYY response to food ingestion was reported in bulimic patients together with a decreased response of ghrelin [110, 111] (Table 2). What might be the role of PYY and ghrelin aberrations in BN? It has been demonstrated that BN patients exhibit impaired CCK secretion [188]. CCK, a satiety factor, is a stimulant of PYY secretion [189]. Hence, depressed PYY levels may result from reduced CCK secretion. Furthermore, both studies have confirmed a negative correlation between PYY increase and ghrelin decrease. The suppression of plasma ghrelin and the increase of plasma PYY_3–36_ after meal may show the compensatory activation of peripheral signals promoting termination of food ingestion. Therefore, Kojima et al. [111] speculate that a gut-hypothalamic pathway involving peripheral hormonal signals, such as ghrelin and PYY, may be related to the pathophysiology of BN. 

### 2.5. CCK

CCK is a member of the gut-brain family of peptide hormones [190]. CCK is secreted by the gastrointestinal system in response to food intake as well as by specialized neurons in the myenteric plexus and the brain [191]. A rise in circulating concentrations of CCK terminates feeding in rats [192]. I.v. CCK infusion decreases hunger and feeding in humans [193]. 

CCK is synthesized as a 115-amino acid prepro-CCK that is cleaved to generate CCK-58. CCK-58 is the largest circulating form of the hormone in humans. From the amino terminus of the peptide, it undergoes sequential proteolytic cleavage generating shorter peptides: CCK-39, CCK-33, CCK-22, CCK-12, and CCK-8. CCK-8 is the smallest fragment with complete biological activity [191]. CCK performs its numerous functions by binding to G-coupled CCK receptors located on the targets organs. Two different receptors have been identified: CCK-1 and CCK-2. CCK-1 receptors are abundant in the gut and in a few discrete brain regions (NTS, the area postrema and the hypothalamus), while CCK-2 receptors are expressed in the cerebral cortex, the hypothalamus, vagal nerve, spinal cord, and gastric mucosa [191]. It was confirmed that CCK released from the small intestine when nutrients enter the duodenum stimulates CCK-1 receptor on sensory fibres of vagal afferents that transfer signals to the NTS in the brainstem [194]; both ghrelin and CCK, after release from the gut, transmit starvation and satiety signals to the brain through the GHS-R 1a and CCK-1 receptors, respectively, located in the vagal afferents. Lesions of the vagus nerve or the NTS abolish the satiety effect of CCK [8]. The satiety hormone CCK activates adrenergic/noradrenergic NTS neurons. Hisadome et al. [195] suggest that epinephrine and NE act as anorectic signals at the level of the NTS. Central administration of CCK also inhibits feeding *via* the activation of CCK-2 receptors which are expressed in the ventromedial and paraventricular hypothalamic nuclei [196]. 

Chen et al. [197] showed that CCK-2 receptor knockout mice had increased body weight but smaller AT mass. Only CCK-2 receptors but not CCK-1 receptors are expressed in AT suggesting that CCK-2 receptors regulate fat metabolism and that CCK-2 receptor may play a role in adipose differentiation. However, Clerc et al. [198] reported that hyperphagia and increased fat deposition were observed in CCK-2 receptor deficient mice. Until now, little has been known about the role of CCK-2 receptors in AT. 

#### 2.5.1. CCK Levels in AN and BN

Some studies in AN patients have found elevations in basal plasma CCK levels [199, 200] as well as increased CCK release following a test meal in anorectic patients [201] (Table 2). Other studies have found that measures of CCK function in AN were similar to or lower than in control subjects [202–204]. 

However, BN patients exhibit impaired CCK secretion [188], and levels of CCK in BN are reduced during symptomatic phases of the disorder and also during a phase of initial recovery [205]. Patients with BN have diminished release of CCK following ingestion of a standardized test meal [187, 206] (Table 2). It has been suggested that the decreased CCK response to a meal may play a role in diminished postingestive satiety observed in BN and that may contribute to the perpetuation and frequent relapse of this disorder [207, 208] (Table 2).

### 2.6. Leptin

Leptin is a 167-amino acid protein known to suppress appetite and regulate energy expenditure, and it suppresses activity of NPY neurons and stimulates pro-opiomelanocortin (POMC)/CART neurons in the arcuate nucleus of the hypothalamus. Leptin is secreted exclusively by adipocytes [209], and leptin has also been found in the stomach [210] and the pituitary gland [211]. Nevertheless, AT remains its main source responsible for 95% of leptin production [212]. Leptin acts through the leptin receptor (OB-R), which is expressed in the hypothalamus and peripheral tissues such as the gut and AT. This ubiquitous distribution of OB-R underlies the pleiotropic roles of leptin [213]. Soluble OB-R represents the major leptin binding activity in human plasma [214]. During nutritional recovery, leptin increases and the OB-R decreases indicating that OB-R could be used as a valid biomarker for nutritional recovery [215]. 

Plasma leptin levels reflect both energy stores and acute energy balance. Circulating leptin levels are positively correlated with BMI and AT mass, and food restriction results in suppression of plasma leptin levels, which can be reversed by refeeding [16]. Peripheral leptin administration reduces food intake resulting in loss of fat mass [216]. It was suggested that leptin-induced increases in energy expenditure may reflect an activation of the SNS [217]. A report by Tang-Christensen et al. [218] support that central leptin administration activates the SNS and that increases plasma NE levels in primates.

Adipocytes possess large numbers of GH receptors, and it was shown that GH directly regulates leptin gene production [219] and that hyperleptinemia may suppress ghrelin secretion [220]. Furthermore, leptin has been shown to play a stimulatory role in GH secretion in rats [221]; however, leptin is likely to exert an inhibitory action on GH secretion *via* a stimulatory effect on hypothalamic somatostatin activity in humans [95], and/or GH hyposecretion might be explained by a resistance to leptin action because hyperleptinemia might contribute to the GH hyposecretion of obese patients [222], and it is suggested that the malnutrition-dependent reduction of leptin levels may play a role in the hypersomatotropism of AN [94, 99, 223]. 

As shown by recent studies, leptin dose dependently inhibits ghrelin transcription *in vitro* [224] and decreases ghrelin release from isolated rat stomach [225]. These findings raised the possibility that hyperleptinemia may suppress ghrelin secretion in obese patients [226]. There is also an opposing relation that ghrelin hypersecretion is in conjunction with hypoleptinemia in AN [94]. The importance of leptin as adiposity signal to the brain is revealed by evidence that leptin inhibits the activity of orexigenic ghrelin-NPY network, whereas low plasma leptin levels upregulate the expression of NPY neurons which coexpress ghrelin receptors [220]. 

Furthermore, the production of leptin is influenced by several regulators, being stimulated by antilipolytic insulin and blood glucose but inhibited by sympathetic activity, lipolytic catecholamines, and FFA [227]. As reported by Frühbeck et al. [228], leptin appears to be involved in the regulation of AT metabolism by both inhibiting lipogenesis [229] and stimulating lipolysis [227]. 

#### 2.6.1. Plasma, Soluble OB-R, and AT Leptin Levels in AN and BN

It was shown that leptin is the major hormone to trigger the adaptation of an organism to food restriction [230]. These findings indicate that the drop in leptin secretion associated with weight loss induced *via* a reduced energy intake is a major trigger underlying adaptation to starvation in AN [231]. In malnourished and underweight AN patients, plasma leptin levels are consistently found to be markedly lower than in normal weight controls [29, 30, 161, 179, 231, 232], and weight recovery in AN is associated with a trend toward, increases in plasma leptin levels [223, 233] (Table 2). In contrast, plasma soluble OB-R level was reported to be increased [232, 234]. This increase may reflect a protective mechanism that decreases free leptin bioavailability and thus further facilitates energy conservation in AN patients [232].

Interestingly, in our study, Dostalova et al. [232] reported significantly reduced plasma leptin levels but normal dialysate leptin concentrations in subcutaneous abdominal AT in AN patients. This finding could be explained by the increased number of smaller adipocytes in subcutaneous abdominal AT leading to a higher number of adipocytes per volume in AN patients when compared with the controls. Another explanation of this may be due to a reduced efficiency of both the SNS [179] and NPY inhibiting adipocyte leptin production in AN. On the other hand, because of reduced volume of subcutaneous abdominal AT, less leptin is secreted into plasma. It has been shown that SNS can exert tonic inhibitory action on leptin secretion and that adrenergic regulation may contribute to rapid decrease both of plasma leptin and insulin levels during exercise in AN and BN patients [6, 7, 30]. 

Recently, Fazeli et al. [99] have shown that administration of supraphysiological recombinant GH in patients with AN leads to significantly decrease in plasma leptin levels when compared with the placebo AN group. 

In normal weight subjects with BN, plasma leptin levels have been reported to be either decreased [235, 236] or normal [234, 237] (Table 2). It has been reported that BN patients with a significantly higher number of daily binge/vomiting episodes hyposecrete leptin in spite of no changes in their BMI [6, 7, 238, 239]. Interestingly, we observed opposite changes in plasma leptin and ghrelin levels during the exercise with Aci administration and in the postexercise recovering phase in both BN patients and healthy women [6, 7, 12, 13, 240]. Plasma soluble OB-R was unaffected in BN patients when compared with the controls [234].

## 3. Conclusions

In AN, there is a characteristic excess of both feeding stimulatory and feeding inhibitory signalling, producing the “mixed” signals for satiety and desire to feed leading to failure of hypothalamic regulatory pathways [241]. AN may be consistent with a state of nutritionally acquired GH resistance [99] and with the mechanism of the state of ghrelin resistance in AN [48, 242]. The ghrelin autoantibodies could alter the feeding regulatory neurocircuitry and eating behavior by changing of the signalling of the hormone ranging from transport to neutralization resulting in the phenomenon of ghrelin resistance in AN patients [48] (Table 1, Figure 2). Very recently, Acres et al. [243] and Hornig and Lipkin [104] hypothesize that AN is an autoimmune disease and may also be associated with major histocompatibility complex (MHC) gene polymorphisms. Indeed, the development of type 1 diabetes in adolescence seems to place girls at risk for the subsequent development of AN and BN [244, 245]. Thus, autoimmune disorders are associated with increased secretion of leptin, whereas AN and BN are conditions of reduced leptin production. Hence, leptin could represent the “missing link” between autoimmunity and nutritional status. Also, BN is associated with an autoimmunity [43]. In BN patients, decreased levels of autoantibodies against serotonin (IgG class) may be involved with the lack of satiety. Also the decreased levels of autoantibodies against dopamine and dopamine-beta-hydroxylase (IgG, and IgM classes) could be implicated in the exaggerated hunger of bulimic patients [43] (Table 1, Figure 2). 

Nevertheless, high ghrelin and low leptin concentrations suggest an orexigenic adaptive mechanism of appetite regulation in response to low food intake in AN [100, 175, 231] (Tables 1 and 2). Differences between central and peripheral secretion of hypothalamic neuropeptides, gut-related peptides, adipocytokines, and the altered cosecretion of hormones with monoamines (NPY-NE; CCK-dopamine; CCK-serotonin; PYY-serotonin) were found in AN and BN [21–23, 37–39, 41, 42]. Indeed, obesity-prone rats have abnormalities in their dopamine system, a key component of hedonic regulation [246], and acutely ill BN subjects have a reduced striatal dopamine transporter availability as well as reduced hypothalamic serotonin transporter availability [247]. Gut hormones, such as PYY and ghrelin, in humans alter brain activation in these corticolimbic areas and higher cortical regions [248, 249]. Thus, patients with BN mainly had an excess of ghrelin with binge eating behavior and decreased anorexigenic signals by neurotransmodulator disturbances [38, 108, 110, 111, 175, 188]. These “mixed” signals could underlie bulimic binge eating behavior in which a relative increase in orexigenic and a decrease in anorexigenic signalling is characteristic (Tables 1 and 2). Ghrelin secretion in AN is higher in its cephalic phase and may eventually facilitate binge eating behavior in bulimic subjects [250]. 

Importantly, the mesolimbic reward system including the ventral striatum and ventral regions of the anterior cingulated cortex and of the orbitofrontal cortex has been proposed to play a pivotal role in the genesis of AN [23, 251] (Figure 1). Indeed, a functional magnetic resonance imaging (fMRI) study showed that the central striatal reward system in AN was hyperactivated upon processing of disease-specific stimuli [252]. Thus, changes in peptidergic neurophysiology occurring in the acute state of an eating disorder may play a pivotal role in the pathophysiology of the disorder by providing a possible link between motivated behavior, reward processes, cognitive functions, and energy balance [253, 254]. Although not consistently, hypoactivation of brain areas was documented in the mesolimbic reward system in women with active AN and weight-restored women with AN [255]. 

Therefore, these observations may contribute to the disruption of AT-gut-brain signalling system and neutralizing autoantibodies in eating disorders (Figures 1 and 2). In recent years, knowledge in the field of food behavior has widely increased, leading to the design of molecules targeted for pharmacological correction of eating disorders and weight control [256]. Also lower dosages of leptin could potentially ameliorate hyperactivity, depression, the metabolic profile, and reproductive function in acutely ill AN patients after weight regain [257]. At present, leptin, ghrelin, GOAT, NPY, tumour necrosis factor-alpha (TNF-*α*) downregulating agents such as dexamethasone and potentially Aci, or their synthetic analogs [6, 7, 13, 56, 256–258] as well as selective serotonin reuptake inhibitors (SSRI) and serotonin NE reuptake inhibitors (SNRI) may be useful agents for the modulation of food intake, especially serotonin-dopamine antagonists (e.g., Olanzapine) which have been found to be effective for treating AN [256, 259]. In the treatment of eating disorders, modified blood-brain barrier in AN and BN is a therapeutic target for delivery of any therapeutics to the central nervous system [103, 104, 260] (Figure 2). The circulating autoantibodies against appetite-regulating neuropeptides and neurotransmitters can be purified and used as monoclonal antibodies in AN and BN. Thus, pharmacologically active monoclonal antibodies would recognize not only the peptide but also the corresponding sequence in the native receptor [261]. Further research is required to investigate the gut-brain-AT orexigenic/anorexigenic agonists or antagonists [262] and the modifications of their pathways with receptors and neutralizing autoantibodies as well as clearance of peptide for potential treatment of eating disorders such as AN and BN in clinical practice [263–265]. Taken together, more data are needed to clarify the etiopathogenesis and pathophysiology of AN and BN. New hopes have arisen through by the recent progress in understanding the immunoneuroendocrine regulation of energy metabolism and feeding behavior because the current long-term pharmacological therapy of anorectic and bulimic patients is almost unsuccessful [266]. Eating disorders and depression are frequently associated. Indeed, an increase of plasma NPY levels may be due to a protective mechanism that prevents the exhaustion of energy reserves in BN and AN patients [6, 14]. Thus, Garcia et al. [267] supported NPY protective role in depression and that decreased plasma levels of NPY autoantibodies (IgG class) are relevant to altered mood while their increased affinities may participate in reduced appetite and body weight in depressive disorder (Table 1, Figure 2). We believe that results of this review may be helpful for better understanding participation of the AT-gut-brain axis peptides and neutralizing autoantibodies on pathophysiology of eating disorders such as AN and BN.

## Figures and Tables

**Table 1:** Summary of the most relevant changes of adipose tissue-gut-brain axis plasma peptides stimulating appetite and autoantibodies against acylated ghrelin before and after refeeding in patients with anorexia nervosa (AN) and bulimia nervosa (BN), and the presence of autoantibodies against neuropeptide Y (NPY) and ghrelin in healthy women and autoantibodies against NPY in depressive disorder. Immunoglobulin (Ig) M, IgG, and IgA classes.

Peptides stimulating hunger and food intake and autoantibodies against peptides and autoantibodies against neurotransmitters	AN		BN	
	Acute phase	Weight restored	Acute phase	Recovered
NPY	→ ↑ ↓	→ ↓	↑ →	→ ↑
NPY (response to test meal)	blunted		↓/blunted	
NPY (response to the exercise)			↑	
Autoantibodies against NPY in healthy women (IgG, IgA)				
Autoantibodies against NPY in depressive disorder (IgG↓)				
Ghrelin	↑	↑ →	→ ↑	→
Ghrelin (response to test meal)	↓/blunted		↓/blunted	
Ghrelin (response to the exercise)			↓	
Autoantibodies against ghrelin in healthy women (IgG, IgA)				
Autoantibodies against acylated ghrelin (IgM)	↓	↑		
Autoantibodies against dopamine, dopamine-beta-hydroxylase, and serotonin (IgG, IgM)			↓	

↑: higher than healthy controls, ↓: lower than healthy controls, and →: not different from healthy controls.

**Table 2:** Summary of the most relevant changes of adipose tissue-gut-brain axis plasma peptides inhibiting appetite in patients with anorexia nervosa (AN) and bulimia nervosa (BN), and the presence of autoantibodies against leptin and peptide YY (PYY) in healthy women. Cholecystokinin (CCK), immunoglobulin (Ig) G, and IgA classes.

Peptides inhibiting hunger and food intake and autoantibodies against peptides	AN		BN	
	Acute phase	Weight restored	Acute phase	Recovered
Leptin	↓	→	→ ↓	→
Leptin (response to test meal)	→		→	
Leptin (response to the exercise)	↓		↓	
Autoantibodies against leptin in healthy women (IgG, IgA)				
CCK	↑ →	→	↓ →	↓ →
CCK (response to test meal)	↑ →	→	↓/blunted	
PYY_3–36_	→ ↑ ↓		→	↑ →
PYY_3–36_ (response to test meal)	↑/blunted	→	↓/blunted/↑	
Autoantibodies against PYY in healthy women (IgG, IgA)				
Obestatin	↑ →	↓	→ ↑	
Obestatin (response to test meal)	↓ →		↓ →	

↑: higher than healthy controls, ↓: lower than healthy controls, and →: not different from healthy controls.

## Abbreviations

AN: Anorexia nervosa
BN: Bulimia nervosa
AT: Adipose tissue
NPY: Neuropeptide Y
PYY: Peptide YY
PP: Pancreatic polypeptide
CCK: Cholecystokinin
NE: Norepinephrine
GH: Growth hormone
GHS: Growth hormone secretagogue
GHS-R: Growth hormone secretagogue receptor
GHRH: Growth-hormone releasing hormone
FGF 21: Fibroblast growth factor 21
FFA: Free fatty acids
OB-R: Leptin receptor
BMI: Body mass index
DPP-IV: Dipeptidyl peptidase IV
CRF_1, 2_-R: Corticotropin-releasing factor receptor subtype 1, 2
CART: Cocaine- and amphetamine-regulated transcript
GOAT: Ghrelin-O-acyl-transferase
3T3-L1: Preadipocytes
GPR39: G-protein-coupled receptor 39
GLP-1: Glucagon-like peptide-1
IGF-1: Insulin growth factor 1
NTS: Nucleus tractus solitarius
SNS: Sympathetic nervous system
Aci: Acipimox
Ig: Immunoglobulin
PPAR-*α*: Peroxisome proliferator-activated receptor-alpha
i.v.: Intravenous
SSRI: Selective serotonin reuptake inhibitors
SNRI: Serotonin norepinephrine reuptake inhibitors
MHC: Major histocompatibility complex
TNF-*α*: Tumour necrosis factor-alpha
*α*-MSH: Alpha-melanocyte-stimulating hormone
HOMA-IR: Homeostasis model assessment of insulin resistance
POMC: Proopiomelanocortin
fMRI: Functional magnetic resonance imaging
PANDAS: Pediatric Autoimmune Neuropsychiatric Disorders Associated with Streptococcal infection
i.e.: In other words.
